# Language enjoyment and emotional engagement: are they distinct or congruent constructs?

**DOI:** 10.3389/fpsyg.2026.1893004

**Published:** 2026-09-01

**Authors:** Fakieh Alrabai, Abdullah A. Alamer

**Affiliations:** 1 Department of English. Faculty of Languages, King Khalid University, Abha, Saudi Arabia; 2 Department of English, College of Art, King Faisal University, Al Ahsa, Saudi Arabia; 3 Graduate School of Education, Stanford University, Stanford, CA, United States

**Keywords:** confirmatory factor analysis, discriminant validity, emotional engagement, foreign language enjoyment, jangle fallacy

## Abstract

Emotional engagement and foreign language enjoyment are two widely studied affective constructs in second language acquisition (SLA) research, yet their empirical distinctiveness has never been formally tested. Substantial item-level overlap between their measurement instruments and consistently high inter-construct correlations raise the possibility of a jangle fallacy: two different labels applied to the same underlying construct. This study subjected both constructs to rigorous psychometric scrutiny using longitudinal data collected from 122 undergraduates at three time points across a second language (L2) semester. Confirmatory factor analysis (CFA) and exploratory factor analysis (EFA) were employed to assess discriminant validity. CFA yielded high latent correlations between the two factors at all three time points (mean *r* = 0.76), and fit indices indicated residual misfit that *post hoc* modifications could not resolve. EFA produced a unidimensional solution at Time 1 and an unstable two-factor structure across waves. Together, these results indicate that the existing instruments do not permit reliable empirical separation of emotional engagement from enjoyment. The findings challenge the treatment of enjoyment as a predictor of emotional engagement, suggest that this relationship may reflect within-construct redundancy, and call for the development of measurement tools that more precisely delineate the boundaries of each construct.

## 1 Introduction

Second language (L2) engagement has been widely recognized as a key factor for successful language learning. This concept is well-grounded within the self-determination theory (SDT) framework that explains human functioning (Zhou et al., 2022). A substantial body of research (e.g., Hiver et al., 2024; Lambert et al., 2017; Mercer, 2019; Noels et al., 2019; Sulis and Mercer, 2025; Wang et al., 2022; Zare and Derakhshan, 2024) has established that engagement is a key factor in fostering motivation for language learning. Engagement not only enhances learners’ interest, enthusiasm, and satisfaction, but also contributes to the development of communicative competence as well as higher achievement and proficiency in the target language. Grounded in the SDT perspective, the concept of language engagement is essentially featured by the learner’s active and sustained behavioral, cognitive, emotional, and social involvement in the language learning process (Lambert and Zhang, 2019; Oga-Baldwin, 2019; Philp and Duchesne, 2016; Sulis, 2022). While the behavioral dimension of learner engagement represents the observed attachment to the language learning process such as participation in classroom activities, successful task completion, asking questions and observable effort and persistence by the learner (Mercer, 2019; Zare and Derakhshan, 2024; Zhang, 2020), the cognitive engagement is often reflected by other behaviors that denote mental investment in learning like focused attention, problem-solving, self-monitoring, and the use of learning strategies to understand and use the language (Philp and Duchesne, 2016; Sulis and Mercer, 2025; Zare and Derakhshan, 2024). Besides the behavioral and cognitive aspects, the emotional (affective) state and experience of the learner such as high degree of interest, enthusiasm, enjoyment, motivation and positive attitudes towards language learning as well as low anxiety remain a core dimension of his/her engagement in the language classroom (Alrabai and Algazzaz, 2024; Alrabai and Algazzaz, 2025; Mercer, 2019; Philp and Duchesne, 2016; Zare and Derakhshan, 2024). While social actions like interaction with teachers and peers, peer and group work, and the harmonious-featured relationship that enhance the sense of relatedness and belonging in the classroom have been acknowledged as a dimension of learner engagement in the language classroom by past research (Kołsut and Szumilas, 2023; Philp and Duchesne, 2016; Svalberg, 2009; Zhou et al., 2023), other views (e.g., Mercer, 2019; Sulis, 2024; Sulis and Mercer, 2025) consider the social component of engagement as being, by default, embedded into the other three dimensions of engagement given that foreign language learning is a fundamentally socially situated process.

## 2 Literature review

### 2.1 Emotional engagement

Of the engagement dimensions elaborated earlier, emotional engagement is of particular interest in the current study, as it captures the affective turn in second language acquisition (SLA) that recognizes the importance of emotions in language learning processes (Alrabai et al., 2025; Hiver et al., 2024). Emotional engagement in language learning stands for a mental state that is usually characterized by positive attitudes, interest, enthusiasm, enjoyment, excitement, relatedness, belonging, and emotional connections with the learning activities, the classroom environment, and teachers and peers in the language classroom (Alrabai and Algazzaz, 2024; Zhou et al., 2023). Besides these features of emotional engagement, one key feature of this dimension of engagement is that it is usually largely manifested in the way that learners experience and express both positive emotions such as enjoyment, interest, pride (Dewaele and Li, 2021; Guo, 2021; Wang et al., 2023) as well as lack of negative emotions such as stress, anxiety, or boredom (Derakhshan et al., 2022; Wang et al., 2023; Zhao and Yang, 2022) in the course of language learning. Like the other dimensions of engagement, enhanced emotional engagement is usually linked to better language learning outcomes. In this regard, emotional engagement has been found to be a key driver of greater participation, increased motivation, greater willingness to communicate, persistence, resilience, and eventually better levels of language achievement and proficiency (Dao and Sato, 2021; Hosseini et al., 2022; Tsang and Dewaele, 2023; Wang et al., 2023; Wang et al., 2024; Zhong et al., 2023).

Learner engagement has been found to be usually shaped by a variety of individual, contextual and emotional factors. The individual variables involve age, gender, and years of study (Derakhshan et al., 2022; Ji et al., 2022; Li et al., 2022;) whereases the environmental and contextual factors include variables like task design, classroom climate, social relationships, and learners’ sense of competence (Derakhshan et al., 2022; Lambert et al., 2017; Li et al., 2022; Sulis, 2022; Sulis and Mercer, 2025; Thao and Macalister, 2023; Zare and Derakhshan, 2024; Zhang, 2020), Besides these factors, learner engagement is influenced by emotional factors like learner emotions of enjoyment and lack of anxiety and boredom (Dao and Sato, 2021; Dewaele and Li, 2021; Hiver et al., 2024; Mercer, 2019; Rotgans and Schmidt, 2017; Skinner et al., 2009; Wang et al., 2023; Zhou et al., 2022), satisfaction of basic psychological needs of autonomy, competence, and relatedness (Jang et al., 2016; Reeve, 2012; Reeve et al., 2018) as well as teacher emotional support (Alrabai and Algazzaz, 2025; Reeve and Cheon, 2021; Sadoughi and Hejazi, 2021; Zhao and Yang, 2022).

Due to the different role that each of such factors play in shaping learner engagement, language engagement has been recognized as being a multifaceted, dynamic, context-dependent, action-oriented, and a fluctuating construct (Alrabai et al., 2025; Dao and Sato, 2021; Guo, 2021; Peng and Jiang, 2021; Sulis, 2022; Sulis and Mercer, 2025; Wang et al., 2023; Zhou et al., 2023). While this multidimensional conceptualization has fostered a richer understanding of how learners participate in language learning, it also presents unique challenges, including the overlap between dimensions and the difficulty of drawing clear conceptual and operational boundaries between engagement and related psychological constructs (Aoyama et al., 2024; Nakamura, 2025). Since engagement is a dynamic, fluctuating concept, students’ emotional engagement often fluctuates over time dependent on many factors such as learner individual differences, classroom climate, teacher and peer emotional support, satisfaction of psychological needs (Alrabai and Algazzaz, 2024; Alrabai and Algazzaz, 2025; Alrabai et al., 2025; Jang et al., 2016; Skinner et al., 2009; Zhou et al., 2023) and the teaching approach (Dao and Sato, 2021; Hosseini et al., 2022; Sulis, 2022). On the top of factors that influence language emotional engagement is learner emotions. In this regard, positive emotions such as enjoyment, pride, and hope have been established as strongly associated with higher emotional engagement, while negative emotions like anxiety, boredom, and shame reduce engagement (Dao and Sato, 2021; Derakhshan and Yin, 2025; Feng and Hong, 2022; Tsang and Dewaele, 2023; Wang et al., 2023). In a particular sense, a big line of empirical and experimental studies showed strong links between language learner enjoyment and emotional engagement (Dewaele and Li, 2021; Dörnyei and Ryan, 2015; Guo, 2021; Fredrickson, 2013; MacIntyre and Mercer, 2014; Oxford, 2016; Wang et al., 2023). This relationship will be discussed in further detail in the following section on language learner enjoyment.

### 2.2 Enjoyment in language learning

Foreign language enjoyment (FLE) is a positive emotional experience that arises when learners’ psychological needs are supported and fulfilled while learning a foreign new language (Zeng, 2021). Enjoyment is considered a core positive emotion driving success in language learning. This emotional state is linked to increased motivation (Ryan and Deci, 2017), greater willingness to communicate, lower negative emotions like anxiety and boredom (Dewaele and Li, 2021; Li and Wei, 2022; Zhang et al., 2024; Zhang et al., 2025), higher satisfaction of basic psychological needs (Alrabai and Algazzaz, 2024; Alrabai and Algazzaz, 2025; Feng et al., 2023), and ultimately better language learning outcomes such as higher academic achievement (Dewaele and Alfawzan, 2018; Dewaele et al., 2024; Tsang and Dewaele, 2023).

Past research has consistently identified three main dimensions of language enjoyment. First, the personal/private enjoyment is the sense of intellectual challenge, accomplishment, and self-fulfillment experienced when achieving personal goals while learning a second language (Dewaele and MacIntyre, 2016). This type of enjoyment is characterized by the sense of personal fulfilment that is derived by the satisfaction of psychological needs and accomplishments (Shirvan et al., 2020) and heightened attention and intellectual focus (Wu and Kabilan, 2025). It is also featured by the feeling of encountering, engaging in, and overcoming optimally challenging tasks (Shirvan et al., 2020; Zhang et al., 2024) as well as the degree of emotion regulation that learners exhibit in the language classroom (Xiao et al., 2024). Second, the social or interpersonal dimension of language enjoyment refers to the fulfilment of social psychological needs in the language classroom via the constructive social interactions and harmonious social bonds with peers and teachers in the classroom (Botes et al., 2021; Jin and Zhang, 2018). The third type of language enjoyment is the teacher-related enjoyment (or teacher appreciation) which pertains to supportive teacher behaviors that promote and sustain language learner enjoyment such as being attentive to students’ emotional states, empathic, emotionally supportive as well as fostering a sense of belonging and building harmonious teacher-student relationships (Alrabai and Algazzaz, 2024; Alrabai and Algazzaz, 2025; Botes et al., 2021; Hejazi and Sadoughi, 2022; Solhi et al., 2024).

The relationship between learner enjoyment and engagement is best explained through the lens of SDT. In light of this theory, consistent evidence (e.g., Alrabai and Algazzaz, 2025; Derakhshan and Fathi, 2023; Tsang and Dewaele, 2023) shows that enjoyment usually emerges as a leading predictor of how emotionally engaged students are in the language class in that higher levels of language learner enjoyment are usually associated with a stronger classroom emotional engagement. Additionally, enjoyment does not only influence student emotional engagement directly but also mediates the relationship between different variables in accounting for this variable. For example, the associations between teacher emotional support, BPN satisfaction and student engagement have been found to be mediated by learner enjoyment (Alrabai et al., 2025).

### 2.3 Enjoyment and emotional engagement: distinct or indistinct?

While enjoyment and emotional engagement are both important affective factors in the process of language learning and are closely linked in such a process as elaborated earlier, the underlying relationship between these two concepts merits an in-depth investigation to assess whether the two constructs are meaningfully distinct. The typical conceptualization of enjoyment indicates that it is a positive emotional state that encompasses the sense of pleasure, satisfaction, or fulfillment while engaging in language learning. Emotional engagement, on the other hand, does not only entail enjoyment in learning but also involves other positive emotions beyond enjoyment like interest and excitement besides the lack of other negative emotions such as anxiety, boredom, and stress in addition to other indicators of involvement in language learning like motivation and active participation (Zhang et al., 2024). This broader scope suggests that enjoyment may be a component of, rather than equivalent to, emotional engagement, and may partly explain why the two constructs are typically defined and operationalized differently (Aoyama et al., 2024; Nakamura, 2025; Zeng, 2021; Zhang et al., 2024; Zhang et al., 2025). Yet several lines of evidence raise questions about whether this conceptual distinction is reflected in the available measurement instruments. First, a close examination of the scales used to measure enjoyment (Botes et al., 2021) and emotional engagement (e.g., Alrabai and Algazzaz, 2024; Kirkpatrick et al., 2025; Skinner et al., 2009; Zhou et al., 2022; Zhou et al., 2023) reveals substantial item-level overlap (see Table 1). When different labels are attached to instruments that share near-identical wording, the result is a jangle fallacy—the erroneous assumption that two constructs are distinct simply because they carry different names (Alharfi and Alamer, 2026; Tay and Jebb, 2018; Oga-Baldwin et al., 2025). Such terminological redundancy raises serious questions about the discriminant validity of enjoyment and emotional engagement as they are currently operationalized in the language learning literature and underscores the need for formal psychometric testing.

**Table 1:** Item similarities between learner enjoyment and emotional engagement in past studies.

Study	Enjoyment	Emotional engagement
Alrabai and Algazzaz (2024); Alrabai and Algazzaz (2025)	1. The teacher is encouraging. 2. The teacher is friendly. 3. The teacher is supportive. 4. I enjoy it. 5. I’ve learned interesting things. 6. I am proud of my accomplishments. 7. We form a tight group. 8. We laugh a lot. 9. We have common ‘legends,’ such as running jokes.	1. This class is fun. 2. When I’m in this class, I feel good. 3. When we work on something in this class, I get involved. 4. When we work on something in this class, I feel interested. 5. I enjoy learning new things in this class.
Botes et al. (2021)	1. The teacher is encouraging. 2. The teacher is friendly. 3. The teacher is supportive. 4. I enjoy it. 5. I’ve learned interesting things. 6. I am proud of my accomplishments. 7. We form a tight group. 8. We laugh a lot. 9. We have common ‘legends,’ such as running jokes.	
Skinner et al. (2009)		1. When I’m in class, I feel good. 2. When we work on something in class, I feel interested. 3. Class is fun. 4. I enjoy learning new things in class. 5. When we work on something in class, I get involved.
Zhou et al. (2022); Zhou et al. (2023)		1. This class is fun. 2. When I’m in this class, I feel good. 3. When we work on something in this class, I get involved. 4. When we work on something in this class, I feel interested. 5. I enjoy learning new things in this class.
Kirkpatrick et al. (2025)		Same scale used by Zhou et al. (2022)

The elusive nature of the engagement construct, and of its affective dimension in particular, has been acknowledged in recent scholarly work. Nakamura (2025) notes that affective engagement, despite being routinely included in multidimensional engagement frameworks, “lacks a clear definition in the literature” and that “the inclusion of emotional experiences within affective engagement raises concerns about distinguishing it from the construct of emotions” (p. 2). This observation points to a fundamental unresolved tension: if the affective dimension of engagement is defined primarily through the presence of positive emotions such as enjoyment and the absence of negative emotions such as anxiety, then the construct becomes difficult to distinguish from the emotions themselves. Aoyama et al. (2024) arrived at a similar conclusion through a different route, documenting in a systematic review of 31 empirical studies that enjoyment was the single most frequently used indicator of the emotional/affective dimension of engagement. Nakamura, in the same colloquium, explicitly raised the question of whether it is “acceptable to regard emotional engagement and the experiential aspect of emotions as synonymous constructs” (Aoyama et al., 2024, p. 2), framing the issue as an open empirical question that the field had not yet resolved. The discussant of that colloquium, Al-Hoorie, reinforced this by invoking jingle-jangle fallacies and calling for engagement measures that “undergo rigorous psychometric validation and demonstrate sufficient discriminant validity from other related constructs” (Aoyama et al., 2024, p. 4). Taken together, these contributions from Nakamura (2025) and Aoyama et al. (2024) signal a growing recognition within the field that the conceptual and operational boundaries of emotional engagement are insufficiently specified, particularly in relation to enjoyment, and that formal psychometric scrutiny is overdue.

Second, evidence in past research (Alrabai and Algazzaz, 2024; Alrabai and Algazzaz, 2025; Guo, 2021; Kirkpatrick et al., 2025; Shakki, 2023) shows that enjoyment very strongly statistically correlates with and predicts emotional engagement in the context of language learning, all likely to the terminology overlap detected in the scales measures these two variables (see Table 2). These strong correlations of enjoyment with and its high predicative power in emotional engagement partly reflect overlapping constructs suggesting that feeling enjoyment is itself a form of emotional engagement. High correlations of this magnitude have been described by earlier studies (e.g., Dörnyei and Ryan, 2015) as exceptionally unusual and consequently raises red discriminant validity flags (Al-Hoorie et al., 2025). The empirical record shows that enjoyment and emotional engagement are not merely correlated; they are correlated at a magnitude that challenges their distinctiveness. Across multiple studies (Alrabai and Algazzaz, 2024, 2025; Guo, 2021; Kirkpatrick et al., 2025; Shakki, 2023), enjoyment has emerged as one of the strongest predictors of emotional engagement in language learning contexts, likely due in part to the item-level overlap in the scales used to measure these two variables (see Table 2). From a psychometric standpoint, correlations approaching or exceeding 0.70 constitute a threat to discriminant validity, and values above.80 are typically interpreted as evidence of convergent rather than discriminant validity (Aubrey et al., 2022; Alamer, 2025; Henseler, 2021; Kline, 2023; Shao et al., 2022; Swami et al., 2023). Several of the correlations reported in Table 2 exceed this threshold, a pattern that earlier methodological work described as exceptionally unusual and a clear discriminant validity red flag (Al-Hoorie et al., 2025; Al-Hoorie et al., 2026; Dörnyei and Ryan, 2015). Taken together, the item-level overlap and the unusually high inter-construct correlations converge on the same conclusion: the two constructs may share more variance than previously assumed, and their empirical separability warrants direct investigation.

**Table 2:** Examples of high positive correlations between learner enjoyment and emotional engagement in past studies.

Study	Scales title (as used in the study)	Degree of correlation
Kirkpatrick et al. (2025)	achievement emotions and emotional engagement	(*r* = 0.80, *p* = 0.000)
Alrabai and Algazzaz (2024)	Enjoyment and emotional engagement	(*r* = 0.755, *p* = 0.000) at T1, (*r* = 0.778, *p* = 0.000) at T2, (*r* = 0.823, *p* = 0.000) at T3.
Alrabai and Algazzaz (2025)	Enjoyment and emotional engagement	(*r* = 0.760, *p* = 0.000) at T1, (*r* = 0.867, *p* = 0.000) at T2, (*r* = 0.815, *p* = 0.000) at T3.

A further concern is that the emotional engagement measures reviewed above have not been subjected to systematic psychometric validation against enjoyment scales. Without such validation, it is impossible to determine whether any observed differences between the constructs reflect genuine divergence or measurement artifact. Addressing this gap is especially timely given the broader call in the field for empirical consolidation of overlapping constructs and greater terminological precision (Al-Hoorie et al., 2025; Al-Hoorie et al., 2026; Oga-Baldwin et al., 2025). For instance, Al-Hoorie et al. (2026) recently examined whether the ideal L2 self and ability beliefs constitute empirically distinct constructs and found that the two could not be reliably separated, illustrating how theoretically differentiated constructs can collapse under psychometric scrutiny. To the best of our knowledge, no prior study has directly tested the discriminant validity of enjoyment and emotional engagement using experimentally collected data. The present study fills this gap by submitting both constructs to rigorous psychometric scrutiny, with the aim of clarifying whether they represent a single unified construct or two empirically separable dimensions of the language learner experience. The findings are expected to contribute to psychometric validation research in educational psychology in general and applied linguistics in particular, and to support the development of more precise theoretical frameworks in these areas.

## 3 Methods

### 3.1 Design

The study used a longitudinal design with data collected at three waves.

### 3.2 Participants and matching approach

122 students from a public university in western Saudi Arabia made up the study’s sample. The participants were undergraduate fourth-year EFL students, with an age range 21 to 24 (Mean *=* 22.58, SD = 0.39). Participants shared similar age ranges, educational backgrounds, cultural backgrounds, and social backgrounds and were enrolled in the same course. At the time of the study, students had been learning English for roughly 12–15 years and demonstrated average level of EFL proficiency as established by the results of the exit exam conducted by their institution (average score = 73 out of 100).

### 3.3 Measures

#### 3.3.1 Questionnaire

An online survey that was created for measuring students’ perceptions of the study variables at Times 1, 2, and 3, which are three separate intervals of time. Language learning enjoyment was assessed using nine items, broken down equally into three subscales: personal enjoyment, instructor appreciation, and social enjoyment (Botes et al., 2021). Five items taken from a study by Skinner et al. (2009) were used to measure emotional engagement in the language classroom. Appendix 1 contains the questionnaire items used in the present study. Respondents were asked to score each statement on a five-point Likert scale, which ranged from “extremely untrue” to “extremely true.” Since there were no negatively-worded items in the survey, higher survey item scores accordingly indicated a higher level of learner enjoyment and emotional engagement. The questionnaire was originally created in English, but it was translated into Arabic, the participants’ native tongue, to reduce any potential impact of inadequate English skills on precise responses. Two independent subject matter experts who were fluent in both Arabic and English worked on the translation. Bilingual experts then performed a back translation from Arabic to English. A team of subject-matter experts reviewed the back-translated scale with the original to check for accuracy, fixing any lingering problems or differences between the two instruments. 48 EFL students and 4 teachers who shared similar characteristics with the main study population participated in a four-week pilot study prior to the main study. Both teachers and students were asked for input on this step about the relevance, consistency, and accuracy of the questionnaire items. Based on this feedback, specific modifications and adjustments were made to the measurements’ components in order to enhance their readability, consistency, and clarity.

### 3.4 Data collection

Students voluntarily provided their informed consent to participate prior to the start of study. All other ethical requirements, such as receiving authorization from the organization where the study was conducted, were properly granted. These thorough efforts made sure that the study met the ethics of research integrity, participant rights, and strict ethical standards.

Three time points were used to collect data: Time 1 during the second week of the semester, Time 2 during the seventh week, and Time 3 during the last week of the semester. Time 1 and Time 2 were separated by 5 weeks, and Time 2 and Time 3 by 5 weeks. Students answered a questionnaire at their classes. The link to the survey was distributed to the students, and a researcher was on hand to answer any questions they had. It took the respondents about 10 to 20 min to finish and submit the survey when they accessed it on their mobile or other electronic devices.

### 3.5 Data analysis

Confirmatory factor analysis (CFA), a statistical method belonging to structural equation modeling (SEM), was employed in this study to comprehensively analyze the relationships between emotional engagement and the emotion of enjoyment. As such, there are not structural models (effects) in our analysis. Since CFA involves assessing measurement models, researchers evaluate the factor structure of the variables. Following the guidelines in SEM, when ordinal data is involved (i.e., data with a limited range of responses), such as when there are five response categories, but all participants only selected four, and there is nonnormality (Kurtosis and or Skewness exceeded −2/+2), we opt for using Diagonally Weighted Least Squares (DWLS) estimator (DeVellis and Thorpe, 2022; Kline, 2023; Wang and Wang, 2020). Furthermore, we employed EFA with oblique rotation (oblimin) to examine the item-level functioning when CFA yielded unstable results.

In this study, we used *Jamovi* (The Jamovi Project, 2026), an interface software that handle both CFA and EFA. In CFA, we estimated the correlations within CFA (between the factors) and confidence intervals (CI; 95%) using 1,000 bootstrap procedure was used. CI provides us with a range of numbers around a point estimate of the correlation (*r* value), within which, with a certain prespecified level of confidence (usually 95%), we believe the true parameter lies. No missing data observed in our data, and no treatment was needed for that. Our sample size satisfies a simple two factors CFA (Alamer, 2025) for each wave.

## 4 Results

### 4.1 Results from the CFA

As explained in a previous section, we have three-time points data where emotional engagement and the emotion of enjoyment were assessed. Accordingly, we had three CFAs for each wave of the data. These are presented in Table 3.

**Table 3:** Results of the three CFAs for emotional engagement and the emotion of enjoyment.

CFA model	*χ* ^2^	*df*	*p*	SRMR	RMSEA	RMSEA 90% CI	CFI	TLI
Time 1	61.54	75	0.868	0.08	0.00	[0.00, 0.03]	1.00	1.00
Time 2	95.18	75	0.058	0.09	0.05	[0.00, 0.07]	0.99	0.99
Time 3	93.27	75	0.075	0.09	0.05	[0.00, 0.07]	0.99	0.99

Generally, the three CFAs resulted in well-fitted models, except for SRMR, which we will discuss later. Based on these findings, we examined the factor correlations in Table 4.

**Table 4:** Correlation between emotional engagement and the emotion of enjoyment based on the CFA at three time points.

CFA model	*r*	CI 95%	*p*
Emotional engagement and emotion Time 1	0.91	[0.85, 0.97]	<0.001
Emotional engagement and emotion Time 2	0.59	[0.29, 0.87]	<0.001
Emotional engagement and emotion Time 3	0.79	[0.67, 0.91]	<0.001

#### 4.1.1 Comments on the results of CFA

Across the three CFAs, the SRMR values of 0.08–0.09 indicate noticeable residual misfit, suggesting that the specified two-factor model does not fully reproduce the observed covariance structure and that some inter-item or inter-factor relations remain misspecified. In addition, the consistently high latent correlations between emotional engagement and enjoyment (mean r=.76 across the three time points) point to limited discriminant validity and imply that the two constructs share a substantial proportion of their variance.

Although we consulted modification indices and freed theoretically defensible parameters, these *post hoc* adjustments yielded only marginal improvements in global fit and did not appreciably reduce the overlap between the factors, raising concerns about capitalizing on chance rather than resolving the core measurement problem. Taken together, the CFA results therefore portray a measurement structure in which emotional engagement and enjoyment behave more like strongly overlapping facets of a broader positive emotional experience than clearly separable latent variables, which motivated the use of EFA to probe the underlying dimensionality and item functioning in a less constrained manner.

### 4.2 Results from the EFA

It was hypothesized if CFA exhibited substantial correlation between the two factors, EFA may present different perspective about the data. The EFA of the three time points are reported in Table 5.

**Table 5:** EFA of the items of emotional engagement and the emotion of enjoyment at three time points.

Item	T1		T2			T3		
	F1	U	F1	F2	U	F1	F2	U
enjoy1	0.85	0.28	0.75	—	0.54	0.74	—	0.50
enjoy2	0.79	0.38	0.46	—	0.68	—	0.84	0.28
enjoy3	0.74	0.45	—	0.31	0.77	—	0.89	0.24
enjoy4	0.63	0.61	0.33	0.34	0.68	0.32	0.51	0.52
enjoy5	—	0.95	0.71	—	0.25	0.64	—	0.45
enjoy6	—	0.96	0.79	—	0.25	0.73	—	0.36
enjoy7	0.68	0.54	0.84	—	0.31	0.95	—	0.20
enjoy8	0.48	0.77	0.85	—	0.35	0.86	—	0.25
enjoy9	0.63	0.61	0.69	—	0.35	0.70	—	0.51
emo_eng1	0.79	0.37	—	0.65	0.60	0.57	—	0.48
emo_eng2	0.80	0.37	—	0.86	0.23	0.54	—	0.56
emo_eng3	0.72	0.48	—	0.91	0.19	0.31	0.41	0.64
emo_eng4	0.62	0.61	—	0.69	0.50	0.52	—	0.54
emo_eng5	0.75	0.43	—	0.62	0.56	0.51	—	0.66

Exploratory factor analysis factor loadings and uniqueness across three time points.

Enjoy = enjoyment items; emo_eng = emotional engagement items; F1 = Factor 1; F2 = Factor 2; U = Uniqueness. Loadings below 0.30 are suppressed and indicated by —. Factor extraction was performed using principal axis factoring with oblique rotation (oblimin).

#### 4.2.1 Comments on the results of EFA

The EFA results further reinforced the concerns raised by the CFA and showed that the factor structure was unstable across the three waves. At Time 1, the analysis essentially produced a one-factor solution, as all substantive loadings appeared on a single factor, which suggests that enjoyment and emotional engagement were not empirically separable at this stage. In addition, several enjoyment items at T1 showed very high uniqueness values (e.g., enjoy5 = 0.95, enjoy6 = 0.96, enjoy8 = 0.77), indicating that some items were not well explained by the extracted factor structure. Although Time 2 was more consistent with a two-factor pattern, the solution was still not clean because at least one item (enjoy4) cross-loaded almost equally on both factors (0.33 and 0.34), which points to conceptual overlap and weak simple structure. The problems became even more evident at Time 3, where the pattern was messier, with clear cross-loadings for enjoy4 (0.32 and 0.51) and emo_eng3 (0.31 and 0.41), alongside several emotional engagement items loading on the enjoyment-related factor, thereby blurring the distinction between the two constructs. Taken together, these EFA findings suggest that the measurement structure lacks stability over time and does not provide strong support for a clear and consistent separation between enjoyment and emotional engagement in the present data.

## 5 Discussion

The present study investigated the discriminant validity of emotional engagement and enjoyment as they are currently operationalized in the language learning literature. Drawing on longitudinal data collected across three time points, we employed both CFA and EFA to assess whether the two constructs are empirically separable. The findings converge on a clear conclusion: the measurement instruments commonly used to assess emotional engagement and enjoyment do not reliably distinguish between the two constructs. This outcome aligns with the conceptual and item-level overlap documented in the literature review (see Table 1) and corroborates the concerns raised by earlier methodological work about terminological redundancy and construct complexity in the field (Al-Hoorie et al., 2025; Al-Hoorie et al., 2026; Aoyama et al., 2024; Nakamura, 2025; Oga-Baldwin et al., 2025).

The CFA results revealed that, although the two-factor models achieved acceptable global fit at each time point, the residual misfit indicated by the SRMR values and the consistently high latent correlations between the two factors painted a more concerning picture. The magnitude of these inter-factor correlations is consistent with the pattern of exceptionally high bivariate correlations reported in prior studies (Alrabai and Algazzaz, 2024, 2025; Guo, 2021; Kirkpatrick et al., 2025; Shakki, 2023), which earlier methodological scholarship described as a clear discriminant validity red flag (Al-Hoorie et al., 2025; Shao et al., 2022). When latent correlations reach this magnitude, the two constructs share a substantial proportion of their variance, suggesting that what is labeled “emotional engagement” in one instrument captures much the same psychological content as what is labeled “enjoyment” in another (Alamer, 2025; Henseler, 2021; Kline, 2023). These findings are consistent with the jangle fallacy concern raised in the literature review: different labels have been attached to instruments that share near-identical wording (Alharfi and Alamer, 2026; Tay and Jebb, 2018; Oga-Baldwin et al., 2025), and the CFA results confirm that this terminological overlap translates into empirical non-separability.

Importantly, the attempts to improve the two-factor model through modification indices and theoretically defensible parameter adjustments yielded only marginal gains in fit and did not reduce the overlap between the factors. This finding suggests that the discriminant validity problem is not simply an artifact of model misspecification but rather reflects a fundamental measurement issue: the item pools used to operationalize emotional engagement and enjoyment are tapping into the same underlying construct. This conclusion is further reinforced by the observation that several of the emotional engagement items (e.g., “This class is fun,” “I enjoy learning new things in this class”) are semantically interchangeable with the enjoyment items, a problem that has been previously documented in the literature but not, until now, subjected to formal psychometric testing. Independently, Nakamura (2025) identified this same issue from a conceptual standpoint, noting that the inclusion of emotional experiences within affective engagement raises concerns about distinguishing it from the construct of emotions. The present CFA findings provide the first direct empirical confirmation of those concerns, demonstrating that the overlap is not merely conceptual but is embedded in the measurement structure itself.

The EFA results further corroborated the CFA findings and provided additional evidence of construct inseparability. At Time 1, all items loaded onto a single factor, yielding a unidimensional solution that is incompatible with a two-construct framework. This one-factor outcome directly challenges the assumption, embedded in much of the existing literature (e.g., Alrabai and Algazzaz, 2024; Derakhshan and Fathi, 2023; Kirkpatrick et al., 2025; Tsang and Dewaele, 2023), that enjoyment is a predictor of emotional engagement rather than a component of it. If the two constructs were genuinely distinct, the EFA should have recovered two clearly separable factors at each measurement occasion; the failure to do so at Time 1 suggests that, at least under certain conditions, learners do not distinguish between feeling enjoyment and being emotionally engaged.

The instability of the factor structure across the three waves is particularly telling in a longitudinal design: if emotional engagement and enjoyment were robustly distinct constructs, their factor separation should be replicable across time points. Instead, the present data show that the degree of separation fluctuates, a pattern that is more consistent with two labels for overlapping variance than with two genuinely independent constructs. This temporal instability echoes broader concerns in the field about the dynamic and context-dependent nature of engagement and the recognized difficulty of drawing clean boundaries between its affective dimension and discrete emotions (Alrabai et al., 2025; Aoyama et al., 2024; Dao and Sato, 2021; Guo, 2021; Nakamura, 2025; Sulis and Mercer, 2025; Zhou et al., 2023), but extends them by suggesting that the measurement instruments themselves may be contributing to the apparent fluctuation.

The high uniqueness values observed for several enjoyment items at Time 1 also merit attention. When items exhibit high uniqueness, they are poorly explained by the extracted factor structure, which may indicate that those items tap into content not shared with the rest of the scale. In the present context, the social enjoyment items (e.g., “We form a tight group,” “We laugh a lot”) stood out in this regard, possibly because they capture a relational dimension that is conceptually closer to social engagement than to the affective core of enjoyment. This observation resonates with the three-dimensional conceptualization of enjoyment proposed by Dewaele and MacIntyre (2016) and validated by Botes et al. (2021), in which personal, social, and teacher-related enjoyment are treated as distinct facets. The present findings suggest that, while personal enjoyment overlaps substantially with emotional engagement, the social and teacher-related facets may be more empirically distinguishable, a possibility that warrants targeted investigation in future research.

Taken together, the CFA and EFA results converge on a single conclusion: the discriminant validity of emotional engagement from enjoyment, as these constructs are currently measured, cannot be established with confidence. This finding has significant implications for how the field conceptualizes, operationalizes, and studies these two variables. It also directly answers the open empirical question posed by Aoyama et al. (2024): whether it is acceptable to regard emotional engagement and the experiential aspect of emotions as synonymous constructs. The present data suggest the answer is, at minimum, that the existing instruments do not permit a reliable distinction between them. If emotional engagement and enjoyment cannot be reliably separated at the measurement level, then studies that position enjoyment as a predictor of emotional engagement (e.g., Alrabai and Algazzaz, 2024, 2025; Derakhshan and Fathi, 2023; Guo, 2021; Kirkpatrick et al., 2025) may be modeling a relationship between two indicators of the same underlying construct rather than a causal pathway between two distinct constructs. This interpretive concern parallels the findings of Al-Hoorie et al. (2026), who demonstrated that the ideal L2 self and ability beliefs collapsed under psychometric scrutiny despite being treated as theoretically distinct in prior research.

Our findings challenge the discriminant validity of emotional engagement from enjoyment and point to two possible resolutions. The first is to reconsider the status of emotional engagement as a standalone subscale within multidimensional engagement frameworks. If the emotional engagement subscale, as currently operationalized, is empirically indistinguishable from enjoyment, then retaining it as a separate dimension risks inflating the dimensionality of engagement models without adding unique explanatory power. Researchers may therefore consider either dropping emotional engagement from the engagement subscales or, at the very least, reconceptualizing it in ways that create clearer empirical boundaries from enjoyment. The second resolution is to acknowledge that enjoyment functions as an emotion that is, in practice, the primary affective content of what has been labeled emotional engagement. Under this view, emotional engagement would not be a separate construct but rather a broader label for the experience of positive emotions, principally enjoyment, in the language classroom. This position is partly consistent with Aoyama et al. (2024) suggestion that the overlaps observed across engagement dimensions and between engagement and related constructs may reflect the real complexities of learner engagement rather than simple measurement error; however, the present findings add that those complexities need not be preserved at the cost of discriminant validity, and that a clearer conceptual architecture is both necessary and achievable. This reframing would align with the perspective articulated in the Introduction, in which emotional engagement is characterized by the experience and expression of positive emotions (Alrabai and Algazzaz, 2024; Zhou et al., 2023), and would resolve the measurement redundancy documented in the present study.

## 6 Directions for future research

The findings of the present study underscore the need for researchers to exercise careful attention when dealing with emotional engagement and enjoyment as separate constructs. The substantial empirical overlap documented here does not imply that the two concepts are theoretically identical; rather, it signals that the current measurement instruments fail to capture whatever distinction may exist between them. Future research should therefore prioritize the development and validation of new measurement tools that more precisely delineate the boundaries of each construct. Specifically, if emotional engagement is to be retained as a distinct dimension, its operationalization must move beyond items that simply ask whether learners feel good, enjoy learning, or find the class fun, as such items are, by definition, enjoyment items. Instead, emotional engagement scales should incorporate indicators of the broader affective repertoire theorized to constitute this construct, including interest, excitement, belonging, relatedness, and the absence of negative emotions such as anxiety and boredom (Zhang et al., 2024; Zhou et al., 2023), in ways that do not overlap with established enjoyment measures.

## 7 Conclusion

The present study examined whether emotional engagement and enjoyment are empirically separable when subjected to rigorous psychometric scrutiny. Drawing on longitudinal data across three time points and applying both CFA and EFA, we found that the two constructs could not be reliably distinguished: inter-factor correlations were consistently high, the EFA yielded an essentially unidimensional solution at Time 1, and the factor structure was unstable across waves. These results provide the first direct empirical answer to the question raised by Nakamura in the Aoyama et al. (2024) colloquium—whether it is acceptable to regard emotional engagement and the experiential dimension of emotions as synonymous constructs—and suggest that the existing instruments do not permit a reliable distinction between them. Theoretically, what the field has treated as a predictor-outcome relationship between enjoyment and emotional engagement may in fact be a within-construct association between two operationally equivalent measures. This extends the growing literature on construct redundancy in applied linguistics (Al-Hoorie et al., 2026; Oga-Baldwin et al., 2025) and corroborates Nakamura's (2025) observation that the affective dimension of engagement lacks a clear definition and risks conflation with the emotions it is meant to incorporate.

These findings carry direct implications for scale development, research design, and theory. Scales whose emotional engagement items are semantically interchangeable with enjoyment items will not yield interpretable discriminant validity evidence regardless of the method applied; researchers should therefore treat item content as a critical design variable. If the two constructs are, in practice, empirically indistinguishable, models that position enjoyment as a predictor and emotional engagement as an outcome may be modeling the same construct twice. A more parsimonious approach would be to treat enjoyment as the core affective indicator of emotional engagement and to reserve the engagement label for genuinely distinct dimensions of the learner experience, such as emotional regulation, affective resilience, and the dynamic management of negative emotions (Xiao et al., 2024; Zhang et al., 2024). Until clearer conceptual and measurement boundaries are established, the field risks building an empirical record on a shaky terminological foundation, and we hope the present findings prompt a productive reexamination of how these constructs are conceptualized, measured, and interpreted.

## Data Availability

The raw data supporting the conclusions of this article will be made available by the authors, without undue reservation.

## Appendix 1

### Students’ questionnaire

Enjoyment:

1. The teacher is encouraging.
2. The teacher is friendly.
3. The teacher is supportive.
4. I enjoy it.
5. I’ve learned interesting things.
6. I am proud of my accomplishments.
7. We form a tight group.
8. We laugh a lot.
9. We have common ‘legends,’ such as running jokes.

Emotional engagement:

1. This class is fun.
2. When I’m in this class, I feel good.
3. When we work on something in this class, I get involved.
4. When we work on something in this class, I feel interested.
5. I enjoy learning new things in this class.
